# Novel Plant-Associated *Acidobacteria* Promotes Growth of Common Floating Aquatic Plants, Duckweeds

**DOI:** 10.3390/microorganisms9061133

**Published:** 2021-05-24

**Authors:** Yasuko Yoneda, Kyosuke Yamamoto, Ayaka Makino, Yasuhiro Tanaka, Xian-Ying Meng, Junko Hashimoto, Kazuo Shin-ya, Noriyuki Satoh, Manabu Fujie, Tadashi Toyama, Kazuhiro Mori, Michihiko Ike, Masaaki Morikawa, Yoichi Kamagata, Hideyuki Tamaki

**Affiliations:** 1 Bioproduction Research Institute, National Institute of Advanced Industrial Science and Technology (AIST), Tsukuba 305-8566, Ibaraki, Japan; yndyasko@gmail.com (Y.Y.); k.yamamoto@aist.go.jp (K.Y.); a-makino@aist.go.jp (A.M.); y-mou@aist.go.jp (X.-Y.M.); y.kamagata@aist.go.jp (Y.K.); 2 Bioproduction Research Institute, AIST, Sapporo 062-8517, Hokkaido, Japan; 3 Department of Environmental Sciences, Faculty of Life and Environmental Sciences, University of Yamanashi, Kofu 400-8510, Yamanashi, Japan; yasuhiro@yamanashi.ac.jp; 4 Japan Biological Informatics Consortium (JBiC), Koto-ku, Tokyo 135-0064, Japan; junko.hashimoto_n2pc@natprodchem.jp; 5 Cellular and Molecular Biotechnology Research Institute, AIST, Koto-ku, Tokyo 135-0064, Japan; k-shinya@aist.go.jp; 6 Okinawa Institute of Science, Technology Graduate University (OIST), Kunigami-gun 904-0495, Okinawa, Japan; satoh32@gmail.com (N.S.); fujie@oist.jp (M.F.); 7 Department of Civil and Environmental Engineering, Faculty of Engineering, University of Yamanashi, Kofu 400-8511, Yamanashi, Japan; ttohyama@yamanashi.ac.jp (T.T.); mori@yamanashi.ac.jp (K.M.); 8 Division of Sustainable Energy and Environmental Engineering, Graduate School of Engineering, Osaka University, Suita 565-0871, Osaka, Japan; ike@see.eng.osaka-u.ac.jp; 9 Graduate School of Environmental Science, Hokkaido University, Sapporo 060-0810, Hokkaido, Japan; morikawa@ees.hokudai.ac.jp; 10 Faculty of Life and Environmental Sciences, University of Tsukuba, Tsukuba 305-8577, Ibaraki, Japan; 11 Microbiology Research Center for Sustainability (MiCS), University of Tsukuba, Tsukuba 305-8572, Ibaraki, Japan; 12 Biotechnology Research Center, The University of Tokyo, Bunkyo-ku, Tokyo 113-0032, Japan

**Keywords:** plant-growth promoting bacteria, *Acidobacteria*, duckweed, host–microbe interaction, co-culture

## Abstract

Duckweeds are small, fast growing, and starch- and protein-rich aquatic plants expected to be a next generation energy crop and an excellent biomaterial for phytoremediation. Despite such an importance, very little is known about duckweed–microbe interactions that would be a key biological factor for efficient industrial utilization of duckweeds. Here we first report the duckweed growth promoting ability of bacterial strains belonging to the phylum *Acidobacteria*, the members of which are known to inhabit soils and terrestrial plants, but their ecological roles and plant–microbe interactions remain largely unclear. Two novel *Acidobacteria* strains, F-183 and TBR-22, were successfully isolated from wild duckweeds and phylogenetically affiliated with subdivision 3 and 6 of the phylum, respectively, based on 16S rRNA gene sequence analysis. In the co-culture experiments with aseptic host plants, the F-183 and TBR-22 strains visibly enhanced growth (frond number) of six duckweed species (subfamily *Lemnoideae*) up to 1.8–5.1 times and 1.6–3.9 times, respectively, compared with uninoculated controls. Intriguingly, both strains also increased the chlorophyll content of the duckweed (*Lemna aequinoctialis*) up to 2.4–2.5 times. Under SEM observation, the F-183 and TBR-22 strains were epiphytic and attached to the surface of duckweed. Taken together, our findings suggest that indigenous plant associated *Acidobacteria* contribute to a healthy growth of their host aquatic plants.

## 1. Introduction

Growth, health, and productivity of plants can be supported and enhanced by beneficial symbiotic bacteria, so-called plant growth promoting bacteria (PGPB) [1,2]. So far, well-known PGPB strains have been found in five bacterial phyla: *Actinobacteria* (e.g., *Streptomyces* and *Frankia*), *Bacteroidetes* (e.g., *Flavobacterium*), *Cyanobacteria* (e.g., *Anabaena*), *Firmicutes* (e.g., *Clostridium* and *Bacillus*), and *Proteobacteria* (e.g., *Rhizobium*, *Burkholderia*, and *Pseudomonas*) [3]. PGPB serve as biofertilizers and/or phytostimulators for host plants; e.g., by providing nutrients (nitrogen fixation, iron, and phosphate solubilization, etc.) and plant hormones (indole acetic acid [IAA] production, etc.). In particular, PGPB have long been studied extensively in the agriculture research field, which harnesses PGPB in rhizosphere and phyllosphere to upregulate productivity of crops and valuable terrestrial plants [4].

Duckweeds (subfamily *Lemnoideae*, formerly known as *Lemnaceae*) are common small aquatic plants, which harbor unique features with great potential as agents for wastewater treatment and phytoremediation, as well as a source of energy biomass, feedstock, and human food [5,6,7]. The subfamily *Lemnoideae* currently comprises five genera—*Spirodela*, *Landoltia*, *Lemna*, *Wolffiella*, and *Wolffia*—of 37 species [8,9]. The plant body structure of duckweeds is quite simple; the plant consists of fronds, floating juvenile tissue lacking stem, and single or multiple root(s), or even no root, depending on the species. Most duckweed species inhabit eutrophic still water, such as a pond, lake, or rice paddy field, and rapidly multiply by vegetative reproduction of daughter fronds that will be separated off from mother fronds. Duckweeds are able to accumulate high amounts of carbohydrates, starch, and proteins up to 18–35%, 21–38%, and 16–42% of dry weight, respectively [6], which are levels comparable to those in legume seeds (40–55% of starch and 25–40% of protein by dry weight) [10]. Being fast-growing and nutrient rich plants, duckweeds have been an excellent model organism in plant biology and biotechnology; however, there are few studies of duckweed PGPB [11,12,13,14,15]. Rather very little is known about PGPB for aquatic plants and their interactions, although terrestrial PGPB have long been paid attention to.

*Acidobacteria* is known to be a rarely cultured bacterial phylum comprising diverse members across 26 subdivisions [16]. Indeed, members of *Acidobacteria* are elusive and fastidious [16,17]. A total of only 61 species have been validly described in this phylum despite its high phylogenetic diversity comparable to the related ‘sister’ phylum *Proteobacteria* consisting of 6,408 species in the NCBI taxonomy database at the time of writing [17,18,19,20,21]. Uncultured members of *Acidobacteria* are widely distributed across environments such as soils, hot springs, mine water, sediments, marine sponges, and terrestrial plants [17,20,22,23,24,25,26,27,28]. In addition, our previous studies also revealed that several uncultured *Acidobacteria* in subdivisions 1, 3, 4, 6, and 8 were present in roots of various aquatic plants, such as *Lythrum anceps*, *Iris pseudacorus*, and *Scirpus juncoides* [29,30]. Contrastively to *Proteobacteria*, which is dominant in plant microbiota and includes some members of well-defined plant symbiotic species [3,31], the eco-physiological roles of plant-associated *Acidobacteria* are poorly understood.

Here we report the isolation of two novel *Acidobacteria* strains from wild duckweeds and their positive effects on duckweed growth and health through co-cultivation experiments. Two novel *Acidobacteria* strains F-183 and TBR-22 belonging to subdivisions 3 and 6, respectively, were successfully isolated from two different duckweeds: *Spirodela polyrhiza* in a pond and *Lemna* sp. in a rice paddy, respectively. Both strains attached to the root and frond surface of duckweeds and clearly enhanced growth (frond number) and chlorophyll production of six different species of duckweeds. As far as we know, our study was the first to demonstrate that *Acidobacteria* promoted growth of the host duckweeds and could contribute to their healthy growth.

## 2. Materials and Methods

### 2.1. Isolation and Identification of Bacteria from Wild Duckweeds

Wild duckweeds (*Lemnoideae* spp.) were collected from ponds, rice paddies, and lotus paddies located in Ibaraki Prefecture (Pref.), Japan, during August 2014 and September 2015. Duckweeds were placed in 50 mL conical tubes and washed by manual shaking with 25 mL of sterile distilled water for five times. Duckweeds were cut into fronds and roots and put separately in 50 mL conical tubes with 10 mL sterile distilled water. Samples were then sonicated to detach duckweed-associated microbes from the plant body, using an ultrasonic homogenizer, sonicstar 85 (AsOne, Osaka, Japan) equipped with a φ3 mm tip. Sonication was performed at a power setting of 40 for 30 s with a 0.5 s/0.5 s on/off interval. Sonicated samples were diluted with sterile distilled water and inoculated on 2.0% (*w*/*v*) agar or 1.5% (*w*/*v*) gellan gum plates of diluted tryptic soy broth (DTS) (pH 7.0) and PE03 (pH 7.0) [32] media. The media were supplemented with 10 mL phosphate buffer (1.0 M, pH 7.0), 0.2 mL vitamin mixture, and 5 mL basal salt solution per liter [33]. CaCl_2_.2H_2_O (1.0 mM, final concentration) was used to solidify the gellan gum. Plates were incubated under dark conditions at 30 °C or 25 °C. Gelling agents were autoclaved separately to the other ingredients in order to enhance culturability, as described previously [34].

After two weeks of incubation, colonies were picked and further streaked on new plates several times for isolation. The genomic DNA was extracted from pure isolates using Extrap soil DNA kit plus, version 2 (Nippon Steel and SUMIKIN Eco-Tech Co., Tokyo, Japan), skipping the beads beating steps and further processed accordingly to the manufacturer’s protocol. The 16S rRNA genes (locus tags: F183_r00030 for the F-183 strain and TBR22_r00010 for the TBR-22 strain) were amplified by PCR using primers Bact 10F (5′-AGAGTTTGATCMTGGCTCAG-3′) and Univ 1492R (5′-TACGGHTACCTTGTTACGACTT-3′) [35]. PCR products were purified using reagent Agencourt AMpure XP (Beckman Coulter Inc., Brea, CA, USA) and applied to a sequencing reaction using BigDye^®^ Terminator v3.1 Cycle Sequencing kit (Thermo Fisher Scientific, Waltham, MA, USA) following manufacturer’s protocols. Sequencing products were purified using the reagent Agencourt CleanSEQ (Beckman Coulter Inc.) and sequenced using the sequencer Applied Biosystems^TM^ 3130*xl* DNA Analyzer (Thermo Fisher Scientific). Sequence data were trimmed manually and analyzed using the MEGA 7.0 software [36]. Purity of strains was checked by direct PCR sequencing of partial 16S rRNA genes as well as microscopic observations of cells.

### 2.2. Preparation of Bacterial Inoculants

We used 200 mL of a liquid medium in 500 mL flasks, capped with silicon resin plugs, for cultivation of bacterial strains. The F-183 strain was cultivated in liquid PE03 medium supplemented with 0.5 g L^−1^ glucose at 30 °C under dark conditions with rotary shaking at 150 rpm. The TBR-22 strain was cultivated statically in liquid DTS medium at 30 °C under dark conditions. A previously known representative PGPB of *Lemnoideae*, *Acinetobacter calcoaeticus* strain P23, was used as a positive control in the PGP trait assays [11]. The P23 strain was cultivated in liquid R2A medium (Nihon Pharmatical Co., Tokyo, Japan) at 25 °C under dark conditions with rotary shaking at 150 rpm. Cells in late exponential phase were harvested by centrifugation at 8000× *g* for 10 min, washed in sterile distilled water four times, and resuspended in the mHoagland solution (modified Hoagland nutrient solution) [37]. Bacterial cell suspensions were finally prepared at OD_600_ = 0.1 in the mHoagland solution for co-cultivation with duckweeds. Heat-inactivated bacterial cells were prepared by pasteurization as follows. Cell suspensions were prepared in a 9φ glass test tube and heated in a water bath at 70 °C for 1 min. Cell suspensions were then diluted in the mHoagland solution as well as the untreated cells. Pasteurized cells were streaked on appropriate culture plates and confirmed inactive by no colony formation over incubation for a month.

### 2.3. Preparation of Aseptic Duckweeds

In this study, we used six species of duckweeds: *Spirodela polyrhiza, Landoltia punctata, Lemna minor, Lemna aequinoctialis, Wolffia arrhiza*, and *Wolffia globosa. S. polyrhiza, L. aequinoctialis*, and *W. globosa* were originally collected in Yamanashi Pref., Japan. *L. punctata* and *L. minor* were collected in Ibaraki and Hokkaido Pref., Japan, respectively. *W. arrhiza* was originally supplied by the late Professor E. Landolt of the Swiss Federal Institute for Technology (ETH) and had been subcultured under aseptic condition for more than twenty years. Strains of aseptic duckweeds were prepared in our laboratory by dipping plants in 70% (*v*/*v*) ethanol (1 min), 0.5% (*v*/*v*) hypochlorous acid with 0.02% (*v*/*v*) Triton X-100 (1 to 4 min, depending on plant size), 70% ethanol (30 s), and in distilled water for several times for rinsing. Treated duckweeds were recovered in the mHoagland solution in a plant chamber at 25 °C under an illumination intensity of 5000 Lux with 16 h/8 h light/dark interval. After recovery, duckweeds were subsampled and put on agar plates of Luria–Bertani (LB; BD, Franklin Lakes, NJ, USA), and in the R2A (BD), DTS, and mHoagland. Duckweed plates were incubated at 25 °C for over two months to confirm no formation of bacterial colonies, the condition that we refer to as ‘aseptic’. Aseptic duckweeds were transferred to a fresh mHoagland solution every two weeks for maintenance.

### 2.4. Co-Cultivation of Bacteria and Duckweeds

To examine the PGP effect of bacterial strains on aseptic duckweeds, duckweed individuals with two fronds were picked and cultivated with bacterial cells suspension in the mHoagland solution (OD_600_ = 0.1), in a plant chamber set under the aforementioned conditions. Conical beakers (200 mL), capped with silicon resin plugs, containing 80 mL medium were used for *Spirodela polyrhiza*. Flat-bottomed glass test tubes (40φ), with conical polypropylene caps, containing 40 mL of medium were used to evaluate the growth of *Landoltia* and *Lemna* species. *Wolffia* species were cultivated in 24-well polystyrene microplates with 2 mL medium. Numbers of fronds were counted in the course of the two-week cultivation. Duckweeds without a bacterial inoculation were used as controls. In addition, to check the possibility that cell debris itself could promote plant growth, duckweed cultures with heat-inactivated bacterial cells were also made and observed. Numbers of fronds were compared with those in the control samples to evaluate plant growth promotion. All experiments were conducted in triplicate. Statistical analysis was performed using Students *t* test.

### 2.5. Microscopic Observations

For fluorescent microscopy, an aseptic duckweed (*L. aequinoctialis*) was co-cultured with bacterial cells (OD_600_ = 0.1) for five days in the mHoagland solution using glass test tubes (40φ) with 40 mL medium. Duckweeds were rinsed gently in distilled water twice and stained to visualize plant-attached bacterial cells using the LIVE/DEAD BacLight bacterial viability kit for microscopy (Thermo Fisher Scientific). Stained duckweeds were mounted on a microscope slide with the mHoagland solution supplemented with 0.5% (wt/vol) agar. Fluorescent microscope (Axio Observer.Z1, Zeiss, Jena, Germany) with the software Axio Vision 4.9.1.0 (Zeiss, Jena, Germany) was used for observation. Observation of plant-attached bacterial cells by scanning electron microscope (SEM) (S-4500, Hitachi, Tokyo, Japan) was performed as described previously [38].

### 2.6. Assays on Bacterial Plant Growth Promoting Properties

Plant growth promoting (PGP) traits (siderophore production, phosphate solubilization, indole acetic acid [IAA] production, and nitrogen fixation) of our *Acidobacteria* strains were examined using well-established conventional methods. Siderophore production was tested using chrome azurol S (CAS) agar (1.5% *w*/*v*) overlaid with LB or R2A (BD, Franklin Lakes, NJ, USA) media [39]. Phosphate solubilization was tested on calcium phytate agar media (PVK, NBRIY, and NBRIP) as described previously [40]. We prepared *Acidobacteria* cell pellets as described above, by centrifugation, and a loopful of each cell pellet was inoculated on those assay plates, using inoculating loops. *Pseudomonas putida* strain UWC1 was used as a positive control strain. IAA concentration was determined colorimetrically with the Salkowski reagent in culture media supplemented with 0, 10, or 100 μg mL^−1^ of l-tryptophan, as previously described [41]. The media used for each strain were as follows: PE03 (supplemented with 0.5 g L^−1^ glucose) and R2A for F-183, DTS and R2A for TBR-22, and R2A for P23. To test the nitrogen fixation potential, the *nifH* gene was detected by PCR using primer sets 19F/407R [42], PolF/PolR [43], and IGK/NDR-1 [44], as described in the references.

### 2.7. Measurement of Chlorophyll

Chlorophyll *a* and *b* were extracted and measured spectrophotometrically as described previously [45]. Fresh duckweeds (approximately 10 fronds) were gently wiped using tissue to remove water and put in 5 mL of *N*,*N*-dimethylformamide prepared in glass vials. Chlorophylls were extracted overnight at 4 °C, under dark conditions, until duckweeds were completely decolorized. Absorbances of the solvents were then measured at wavelengths 663.8, 646.8, and 750 nm to calculate the chlorophyll *a* and *b* concentrations.

### 2.8. Genome Analysis of Isolated Acidobacteria Strains

The whole-genome shotgun sequencing was previously performed using a Miseq system (Illumina, San Diego, CA, USA), and the obtained sequence data were assembled using the Newbler v. 2.9 (Roche Diagnostics, Basel, Switzerland) [46,47]. Gene prediction was performed with the annotation tools Prokka v. 1.13 or v. 1.14.6 [48] and BlastKOALA KEGG service v.2.2 [49]. Manual gene function prediction was performed using the NCBI blastp [50] and Lalign tools [51]. Details are described in Figure S3.

## 3. Results

### 3.1. Isolation of Novel Acidobacteria Strains

**Figure 1:**
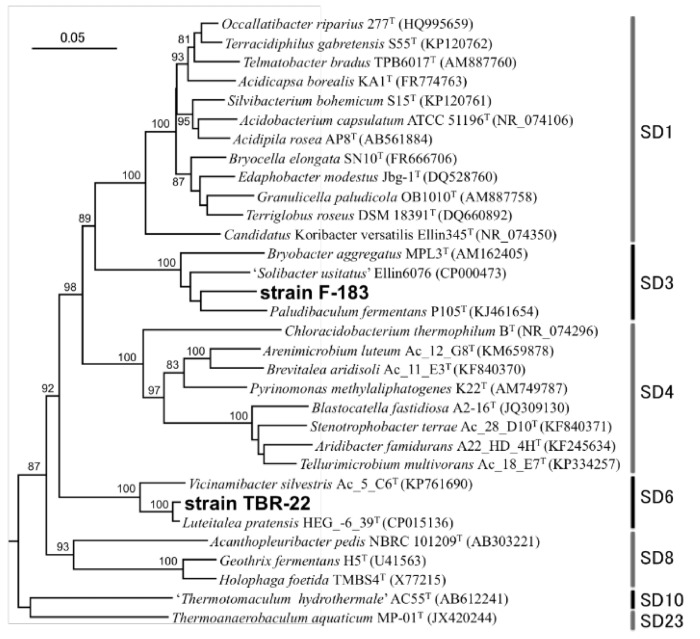
Phylogenetic relationship of the novel isolates and representative *Acidobacteria* species based on the 16S rRNA gene. The tree was constructed by Neighbor-joining methods. *Planctomyces maris* DSM 8797^T^ (AJ231184) and *Rhodopirellula baltica* SH 1 (NR_043384) were used as outgroups. Bootstrap values (>80) were calculated from 1000 replicates. SD, subdivision; bar, 5 nucleotide substitutions per 100 nt.

Two novel *Acidobacteria* strains, F-183 and TBR-22, were isolated from fronds of wild duckweeds, *S. polyrhiza* from a pond and *Lemna* sp. from a rice paddy, respectively, both located in Tsukuba city, Ibaraki, Japan. Almost full lengths of the 16S rRNA genes, 1423 bp for the F-183 strain and 1488 bp for the TBR-22 strain (Supplementary data), were sequenced and subjected to homology search against DDBJ/EMBL/GenBank nr/nt databases using BLASTn. The F-183 strain was moderately related to *Paludibaculum fermentans* isolated from a littoral wetland [52] (93.1% sequence similarity) and *Bryobacter aggregatus* isolated from acidic *Sphagnum* peat bogs [53] (92.2%) belonging to subdivision 3. The closest relative of the TBR-22 strain was *Luteitalea pratensis* HEG_-6_39^T^ isolated from a temperate grassland soil [54] (98.5%) followed by *Vicinamibacter silvestris* Ac_5_C6^T^ isolated from a riparian woodland soil [55] (93.8%) belonging to subdivision 6. In the phylogenetic tree we constructed, the F-183 and TBR-22 strains fell into subdivisions 3 and 6, respectively, with robust bootstrap values (100%) (Figure 1).

### 3.2. Plant Growth Promotion by the Novel Acidobacteria Strains

**Figure 2:**
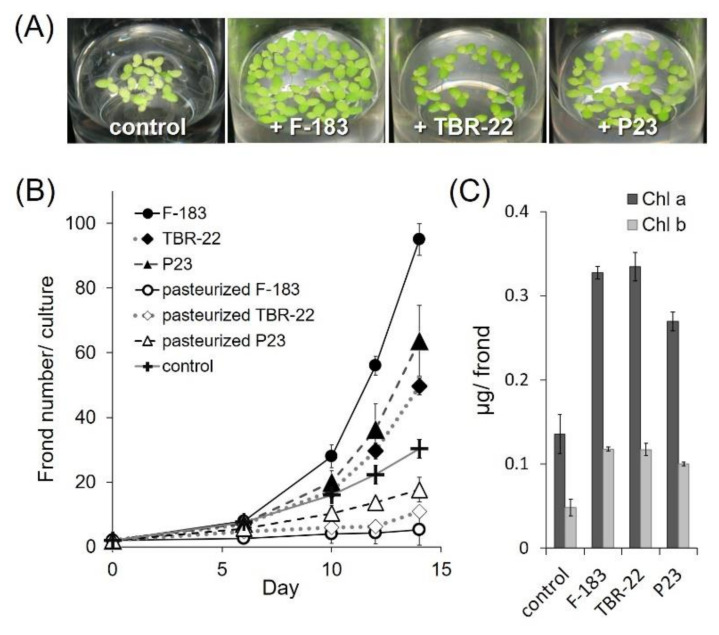
(**A**) Growth of *L. aequinoctialis* co-cultured for 14 days without bacteria (control), with the F-183 strain, the TBR-22 strain, or the P23 strain. (**B**) Growth of *L. aequinoctialis* with active and inactive (pasteurized) bacterial cells. (**C**) Chlorophyll a and chlorophyll b contents of *L. aequinoctialis* after co-cultivation with bacterial strains. Values are expressed as means of three independent experiments. Bars, SD.

To evaluate the PGP ability for duckweeds, we co-cultured the F-183 or TBR-22 strain with aseptic duckweed, *Lemna aequinoctialis*, in the mHoagland solution and examined the increase of the duckweed frond number after two weeks of incubation. The F-183 and TBR-22 strains clearly promoted the growth of *L. aequinoctialis* and led to a 3.1-fold and 1.6-fold increase in the frond number, respectively, compared with uninoculated control (Figure 2A,B). These PGP effects were comparable to that of the known PGPB strain, *Acinetobacter calcoaceticus* P23 [11,12,15,30,56] that multiplied the frond numbers up to 2.1 times. Heat-inactivated cells of the F-183 and TBR-22 strains did not promote the growth of *L. aequinoctialis* at all, indicating that live bacterial cells of the strains were required for plant growth promotion, and the nutrient input derived from heat-inactivated cells by inoculation itself was negligible (Figure 2B).

We further observed that the fronds of *L. aequinoctialis* co-cultured with bacterial strains presented deeper green color, indicating increased chlorophyll contents of the host plants compared with those of the aseptic controls (Figure 2A). Indeed, chlorophyll *a* and *b* contents of the fronds determined at the end point of the incubation increased up to 2.4 times by the F-183 strain, 2.5 times by the TBR-22 strain, and 2.0 times by the P23 strain compared with the uninoculated controls. This indicated that the *Acidobacteria* strains tested here could enhance chlorophyll production in the fronds of *L. aequinoctialis* as well as promote their growth.

**Figure 3:**
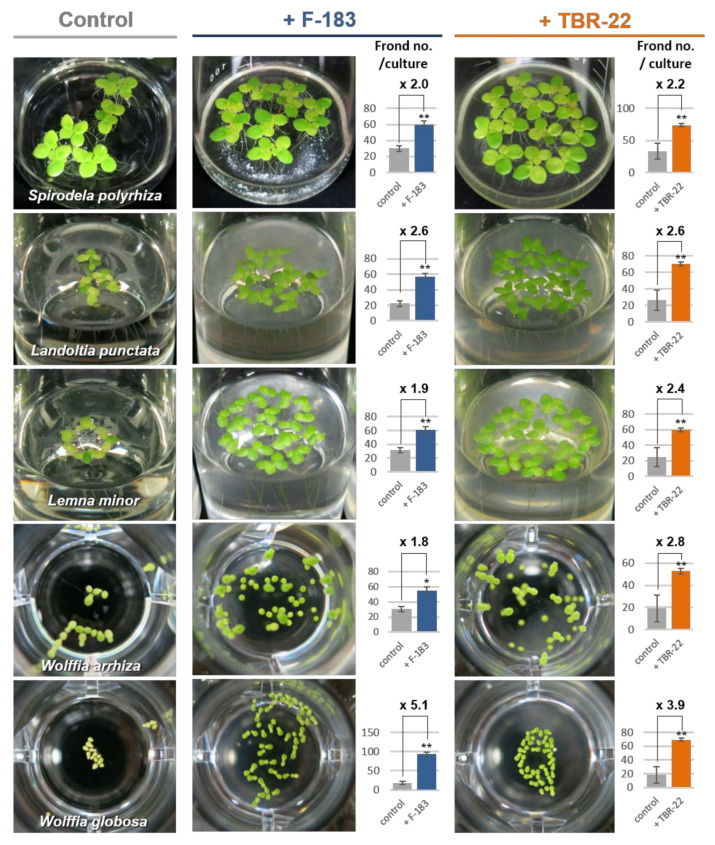
Plant growth promotion effects of the *Acidobacteria* strains F-183 and TBR-22 on duckweed members of the subfamily *Lemnoideae*. Images were taken at day 14 in independent experiments from the quantitative growth promotion assays represented by the bar plots. Frond number was manually counted at day 14 (see Methods). *n* = 3; **, *p* < 0.01; *, *p* < 0.05; bars, SD.

The plant growth promotion by the F-183 and TBR-22 strains was not specific to *L. aequinoctialis* but also observed in various other duckweed hosts (Figure 3, Table S1). The F-183 and TBR-22 strains increased the frond number up to 2.0- and 2.2-fold for *S. polyrhiza*, 2.6- and 2.6-fold for *L. punctata*, 1.9- and 2.4-fold for *L. minor*, 1.8- and 2.8-fold for *W. arrhiza*, and 5.1- and 3.9-fold for *W. globosa*, respectively, compared with corresponding uninoculated controls. During the incubation period, all duckweeds co-cultured with bacteria exhibited a more greenish color of the fronds than the uninoculated control did, indicating an increase of chlorophyll contents as the case of *L. aequinoctialis*.

### 3.3. Acidobacteria Strains Colonization of Duckweed Plant Surface

**Figure 4:**
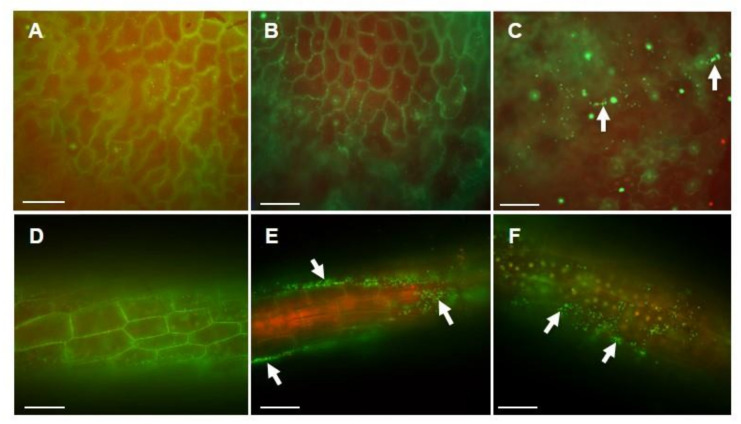
Fluorescent micrographs of live/dead stained fronds (**A**–**C**) and roots (**D**–**F**) of aseptic *L. aequinoctialis* (**A**,**D**) and of *L. aequinoctialis* co-cultured with the F-183 (**B**,**E**) and TBR-22 (**C**,**F**) strains. Live and dead cells are visualized in green and red, respectively. Arrows indicate examples of live stained cells. Bars, 50 μm.

A secure attachment on the plant surface is considered as one of the key factors for PGPB to initiate and sustain a symbiotic plant–microbial association. We investigated the colonization of the duckweed surface by *Acidobacteria* strains by fluorescent microscopic observations. As shown in Figure 4, live cells of the F-183 and TBR-22 strains were observed on the surface of *L. aequinoctialis*, suggesting that both strains could attach to the duckweed surface and colonize it. The F-183 cells were exclusively observed on root surfaces, while the TBR-22 cells were observed on both frond and root surfaces.

**Figure 5:**
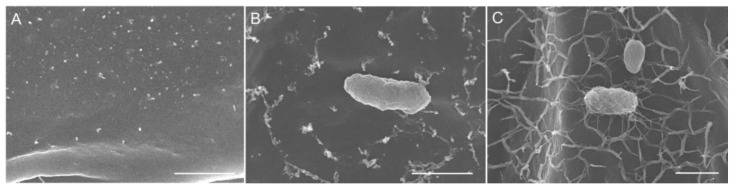
SEM images of *L. aequinoctialis* fronds surfaces of the aseptic control (**A**) and the F-183 (**B**) and TBR-22 (**C**) co-cultures. Bars, 1.0 μm.

When observed with SEM, the F-183 and TBR-22 cells were also found attached on the duckweed surfaces. Notably, network structures produced by the *Acidobacteria* strains were observed on bacterial cells and the frond surfaces when co-cultured with the F-183 or TBR-22 strains, whereas no such structure was observed on the aseptic control (Figure 5). The network structures covered the frond surfaces all over, while they were not observed on the root surfaces in co-cultures with neither F-183 nor TBR-22 (data not shown).

### 3.4. Assays on Bacterial Plant Growth Promoting Traits

The typical traits contributing to plant growth promotion generally found in terrestrial PGPB (siderophore production, phosphate solubilization, IAA production, and nitrogen fixation) were examined in the F-183, TBR-22, and P23 strains. Siderophore production was tested using chrome azurol S (CAS) agar overlaid with Luria–Bertani or R2A agar media for each strain. No obvious orange halo was observed around the colonies of F-183, TBR-22, and P23, whereas a clear halo appeared around a colony of the positive control strain, *Pseudomonas putida* UWC1 (Figure S1). Phosphate solubilization was tested using three agar assay plates (PVK, NBRIY, and NBRIP) for each strain (Figure S2). No apparent halo was formed around the colonies of F-183 and TBR-22 on all three kinds of assay plates tested, while a positive phosphate solubilization was observed in strains P23 and UWC1 in all assays, indicating that the two *Acidobacteria* strains were negative for phosphate solubilization. IAA production was detected in the F-183 strain that had grown in PE03 and R2A media supplemented with 100 μg mL^−1^ of L-tryptophan. Concentrations of IAA were 31 and 13 μg mL^−1^ in PE03 and R2A, respectively. No IAA production was detected in the TBR-22 and P23 strains under any conditions tested. We further found a lack of a key gene for nitrogen fixation (*nifH*) in the genomes of F-183 and TBR-22 by a specific PCR targeting of *nifH*, suggesting that the *Acidobacteria* isolates were not able to fix nitrogen.

### 3.5. PGP-Related Genes in the Genomes of the Isolated Acidobacteria Strains

Draft genome sequences of the F-183 (Genbank accession no. AP024453) and TBR-22 (AP024452) strains were recently obtained [46,47]. To evaluate the PGP potential of these strains, presence of genes related to typical PGP traits was evaluated by using gene annotation pipelines and additional manual annotation (Tables S2 and S3).

Gene sets for biosynthesis of typical siderophores (e.g., *ent*, *pvd*) were not detected in the genomes of F-183 and TBR-22, indicating that these strains were unable to produce typical siderophores. Phosphate solubilization ability of bacteria is mainly attributed to their production and secretion of organic acids [57]. Among many candidates, genes related to gluconic acid metabolism (e.g., *gcd*, PQQ-dependent glucose dehydrogenase; *pqq*, pyrroloquinoline quinone (cofactor) biosynthesis) and alpha-ketogluconic acid metabolism (*gad*, gluconate 2-dehydrogenase) are normally regarded as key genes responsible for the phosphate solubilization ability of PGPB [58]. The genomes of the F-183 and TBR-22 strains harbored genes encoding homologues of glucose dehydrogenase (EC1.1.5.2) and gluconate 2-dehydrogenase (EC1.1.99.3), but a complete *pqq* gene set was not found.

Three major prokaryotic IAA biosynthesis pathways are known, and only a part of these pathways (e.g., conversion of some precursors into IAA: 3-indoleacetonitrile aminohydrolase (EC3.5.5.1), indole-3-acetamide amidohydrolase (EC3.5.1.4), indole-3-acetaldehyde:NAD+ oxidoreductase (EC1.2.1.3) was predicted from both genomes, though syntheses of relevant IAA precursor(s) were not supported (Figure S3A). As the IAA production was experimentally confirmed in the F-183 strain, genes responsible for the production of IAA precursor(s), indole-3-acetamide or indole-3-acetaldehyde, were manually searched using blastp and gene function prediction pipelines. Predicted genes, having high similarity to the known tryptophan 2-monooxygenase (EC1.13.12.3) and tryptamide oxidase (EC1.4.3.4) sequences, which may be involved in the IAA production, were found in the F-183 genome (Figure S3B). The absence of the *nifH* gene was confirmed by the genome survey in both strains, and other key nitrogen fixation genes (*nifDK*, *vntDKGH*, *anfG*) were also not detected, indicating that these strains were incapable of nitrogen fixation.

Other genes or gene sets related to plant-beneficial traits, which are not physiologically evaluated here—such as the *phn* gene cluster (phosphonate metabolism; phosphate release via phosphonate degradation), *budABC* (acetoine/2,3-butanediol synthesis; induced systemic resistance), *nirK* (nitric oxide synthesis; formation of the NO root-branching signal), and *acdS* (1-aminocyclopropane-1-carboxylic acid deamination; degradation of the plant’s ethylene precursor)—were not predicted or only partially predicted from the genome information of the F-183 and TBR-22 strains (Table S4); therefore, contribution of the most conventional PGP traits, except for the IAA production in the F-183 strain, to the observed duckweed growth promotion is likely to be negligible (Figure S4).

## 4. Discussion

Increasing attention to PGPB from both basic and applied viewpoints has driven the recent advances in the relevant research fields [59,60,61]. Nevertheless, our knowledge has mainly been derived from a limited number of phylogenetic groups in the diverse microbial world, and the phylogenetic, functional, and mechanism diversities of PGPB are still largely unknown. Recently, a few *Acidobacteria* strains of subdivision 1, derived from decaying wood, were reported to promote plant growth on a terrestrial well-defined model plant, *Arabidopsis thaliana* [62]. This report added a new phylum to the list of PGPB; however, the ecological significance of such a PGP effect in natural environments is unclear because the observed PGP effect was demonstrated on a model plant. To better understand symbiotic relationships between *Acidobacteria* and plants, it is important to analyze interactions with natural plant hosts, which are established through multiple molecular responses between microbes and plants [63]. In this study, we were successful in isolating novel *Acidobacteria* strains from living wild duckweeds and showed their PGP effect on duckweeds, representing a rare example of symbiotic relationship between *Acidobacteria* and its natural host plants. Our results consolidated that PGPB are distributed in the phylum *Acidobacteria* and potentially widespread within the phylum as new PGPB were found in two distinct subdivisions, 3 and 6 [29,30].

The newly isolated *Acidobacteria* strains, F-183 and TBR-22, exhibited a PGP effect on various *Lemnoideae* species (*S. polyrhiza*, *L. punctata*, *L. minor*, *L. aequinoctialis*, *W. arrhiza*, and *W. globosa*) so the PGP function of F-183 and TBR-22 did not seem to be species specific, at least among the *Lemnoideae* subfamily (Figure 2 and Figure 3). Since chlorophylls represent a limiting factor of photosynthesis and the production of chlorophylls was enhanced by the two strains (Figure 2A), the growth promotion of duckweeds might be partly achieved through enhancing their photosynthesis. The PGP effects on duckweeds by both strains were comparable to that of a previously known representative PGPB of duckweeds, the P23 strain. Among the common PGPB evaluation criteria tested in this study, there was no correlation between the strength of the PGP effect and the number or type of the observed PGP traits: F-183 showed only IAA production, TBR-22 showed nothing positive, and P23 exhibited phosphate solubilization solely. We found that external addition of IAA did not improve the growth of *L. aequinoctialis* (Figure S5, consistent with a previous report describing a negligible effect of IAA on the growth of *L. minor* [64]), suggesting that IAA production did not contribute to the PGP ability of the F-183 strain. On the other hand, it was reported that 79% of endophytic bacterial isolates from *Landoltia*, *Lemna*, *Spirodela*, and *Wolffia* were positive in the Salkowski reagent assay [65]. This result suggested that internal production of IAA might be important for promoting the growth of the duckweed. Ishizawa et al. reported a PGP effect of several aquatic bacterial isolates on *L. minor* and demonstrated that there was no clear-cut correlation between the PGP effect and the possession of conventional PGP traits (phosphate solubilization and production of IAA, siderophore, and HCN) among their PGPB isolates [14]. Besides, the genomes of F-183 and TBR-22 harbored no, or only partial, gene sets for most conventional PGP traits (Figure S4), which was well consistent with the results of the physiological PGP assay (e.g., deficiency of siderophore production and phosphate solubilization). This also indicates that the conventional PGP is not likely to confer the duckweed growth promotion ability on the *Acidobacteria* isolates. In our preliminary experiments, membrane-separated co-culture with the F-183 or TBR-22 strain also enhanced the growth of duckweed (Figure S6), intriguingly suggesting the possibility that diffusible factor(s) produced by the *Acidobacteria* isolates would, at least partly, contribute to the PGP effect, though the underlying mechanisms need to be clarified through identification of the key compound(s) in future studies. A few combinations of aquatic plants and bacterial species have been documented for their symbiotic relationships so far; e.g., nodulating bacteria of water legume [66,67] and *Cyanobacteria* of water fern [68]. Those symbiotic relationships were shown to be mediated by plant hormone production and nitrogen-fixation; however, the exploration of PGPB has recently begun to target aquatic environments, especially duckweeds. Therefore, further exploration of aquatic PGPB and their characterization would lead to discoveries of unknown PGP mechanisms unique to aquatic plant–PGPB relationships.

Coexistence in close proximity (e.g., root colonization) has been considered important for the interaction between terrestrial plants and PGPB [69]. Although this time, the effect of the F-183 and TBR-22 strains on the growth of *L. aequinoctialis* was evaluated in bacterial suspension culture conditions, physical association was indicated as a key factor also in PGP activities on aquatic plants. Physical association might be more important for the PGP activities in aquatic environments, where there can be a constant water flow and higher substrate diffusion rate than in a terrestrial rhizosphere. Intriguingly, we detected network structures, very likely composed of extracellular polymeric substances (EPS), covering bacterial cells and frond surfaces in the co-culture experiment (Figure 4), and they can be responsible for the tight cell attachment and *Acidobacteria* colonization of the plant surfaces. EPS in rhizosphere can alter plant immune response to rhizobia to establish symbiosis [63]. Plenty of bacteria produce EPS for surface attachment and biofilm formation [62,70]. Therefore, EPS produced by *Acidobacteria* itself may have key roles in the adherence to plant surfaces.

Our current study demonstrated the plant growth promotion by plant-associated *Acidobacteria* through co-cultivation with aquatic plants under laboratory conditions. Given that *Acidobacteria* ubiquitously exist in various natural environments and especially have been frequently detected as common soil bacteria [22,71,72,73,74,75,76], our findings not only expanded our knowledge of plant–microbe interactions but also shed light on the ecophysiological aspects and functional roles of this rarely characterized bacteria within natural ecosystems. Further investigation will lead to better understanding of phylogenetic and functional diversities of PGPB for aquatic plants, and to characterizing ecological roles of *Acidobacteria* in aquatic plant microbiota through unveiling the mechanisms behind their aquatic plant growth promotion.

## Supplementary Materials

The following are available online at https://www.mdpi.com/article/10.3390/microorganisms9061133/s1: Figure S1: Detection of siderophore production on CAS agar overlaid with LB medium, Figure S2: Detection of phosphate solubilization on calcium phytate agar plates, Figure S3: Prediction of IAA biosynthesis pathways, Figure S4: Presence/absence of genes related to typical PGP traits in the genomes of the F-183 and TBR-22 strains, Figure S5: Effect of IAA on duckweed growth, Figure S6: PGP effect of the F-183 and TBR-22 strains on duckweed in membrane-separated co-cultivation, Table S1: Plant growth promotion effects of *Acidobacteria* strains F-183 and TBR-22 on duckweed members of subfamily *Lemnoideae*, Table S2: Predicted genes related to PGP traits in the genome of the F-183 strain, Table S3: Predicted genes related to PGP traits in the genome of the TBR-22 strain, Table S4: Predicted genes related to PGP traits in the genomes of the F-183 and TBR-22 strains, Supplementary Data: sequences used for constructing the phylogenetic tree.
