# Supplementary material for: Novel Plant-Associated Acidobacteria Promotes Growth of Common Floating Aquatic Plants, Duckweeds
Source: Microorganisms. 2021 May 24;9(6):1133. doi: 10.3390/microorganisms9061133 (PMC8225144; doi:10.3390/microorganisms9061133)
Supplement: Supplementary file 1 [file microorganisms-09-01133-s001.zip › AcidoPGPB_supplement_Figs_Tables_proof.pdf]

Supplementary Figure S1

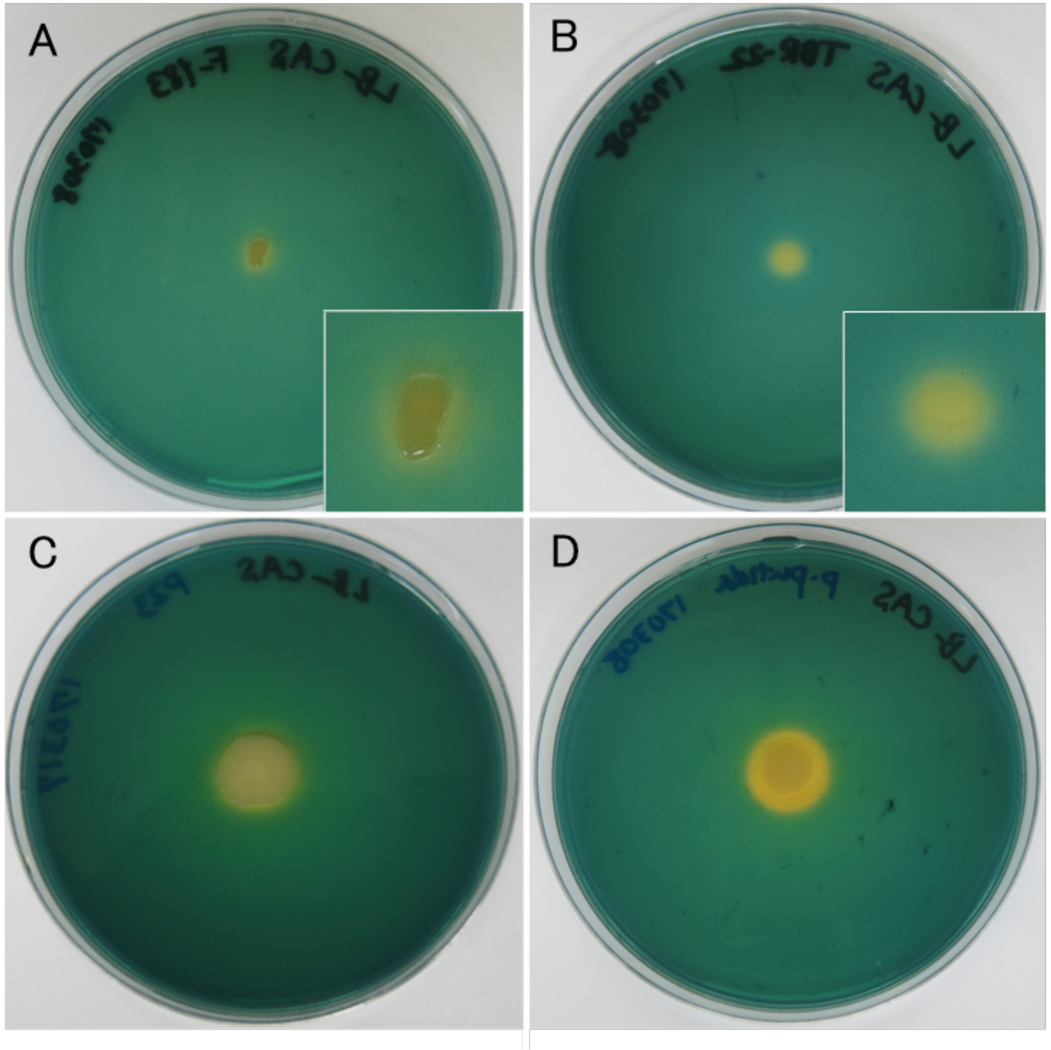

Figure S1. Detection of siderophore production on CAS agar overlaid with LB medium. (A) F-183; (B) TBR-22; (C) P23; and (D) UWC1.

Supplementary Figure S2

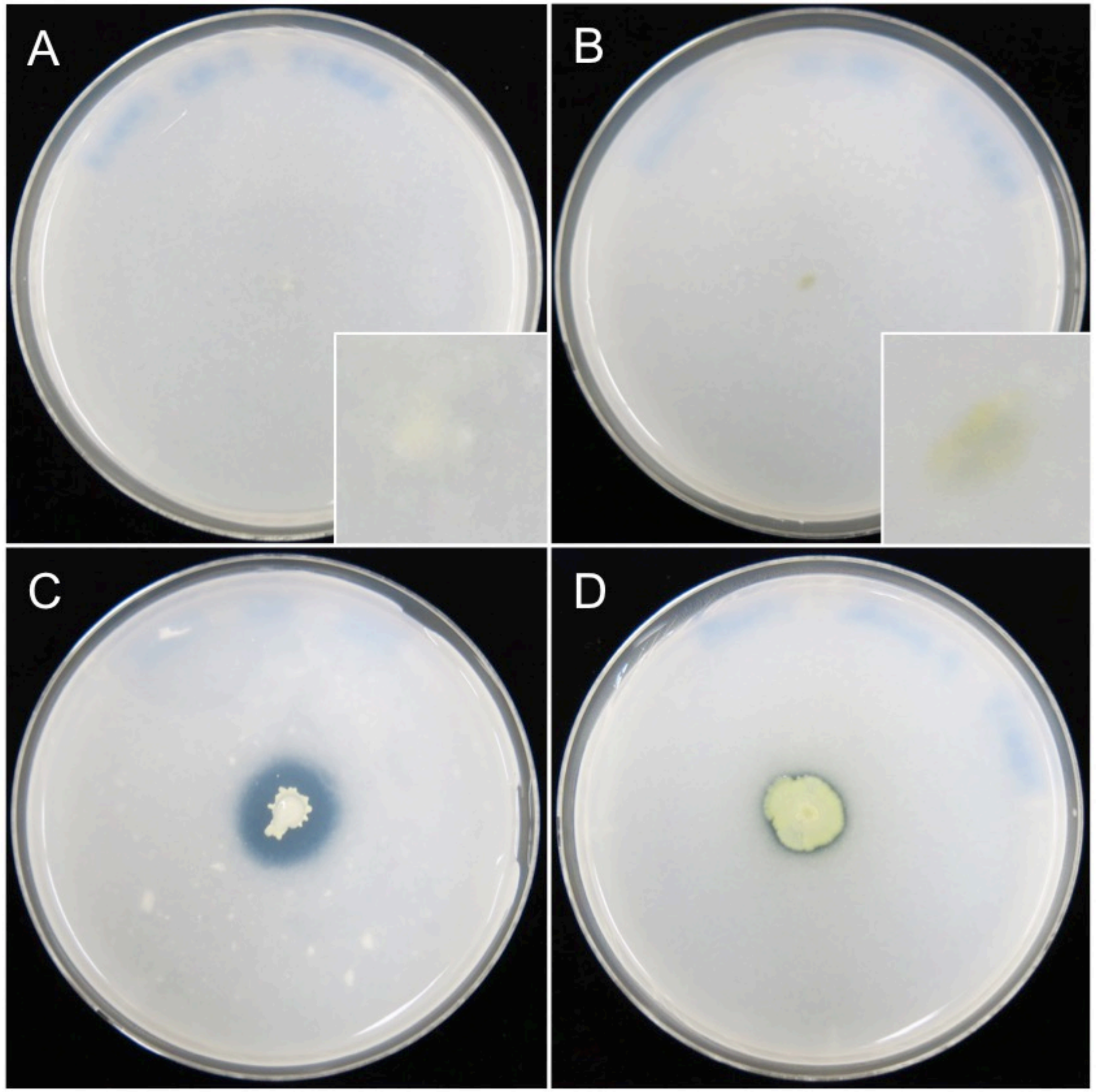

Figure S2. Detection of phosphate solubilization on calcium phytate agar plates. (A) F-183 on NBRIY; (B) TBR-22 on NBRIP; (C) P23 on PVK; (D) UWC1 on NBRIY.

Supplementary Figure S3

(A)

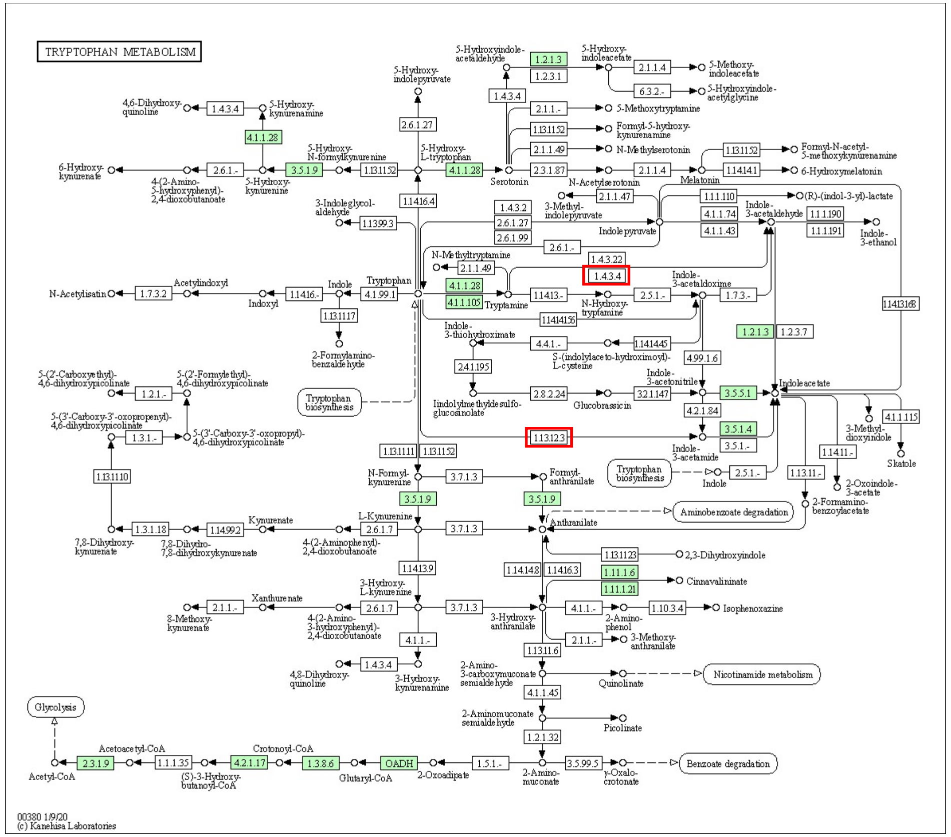

(B)

Prediction of tryptophan 2-monooxygenase [EC1.13.12.3]

NCBI blast search (blastp) against known bacterial tryptophan 2-monooxygenase sequences (1774 seqs) using the F-183 genome sequence as a query.

↓

An ORF (F183_06810 [776944..778215] Xaa-Pro dipeptidase) was found to be a highly similar sequence to tryptophan 2-monooxygenase (top hit = AWL13420.1 Tryptophan 2-monooxygenase [*Saliniradius amylolyticus*], E-value=3.22E-94, 43.77% identity).

Prediction of tryptamide oxidase [EC1.4.3.4]

An ORF (F183_30280 [3507526..3509475]) annotated as Primary amine oxidase was found in the F-183 genome.

↓

F183_30280 had 30.6% identity (Lalign [https://embnet.vital-it.ch/software/LALIGN_form.html]) with Primary amine oxidase O [*Klebsiella aerogenes*], which is active on tyramine, tryptamine, beta-phenethylamine and dopamine (https://www.uniprot.org/uniprot/P49250).

Figure S3. Prediction of IAA biosynthesis pathways. (A) KEGG pathway map (Tryptophan metabolism). Map was generated KEGG mapper using ko file as a query. Green boxes indicate enzymes predicted to exist in the F-183 genome. Red boxes indicate key enzymes which were not predicted by BlastKOALA. (B) Manual function prediction workflow of genes related to production of intermediates in IAA biosynthesis pathways.

Supplementary Figure S4

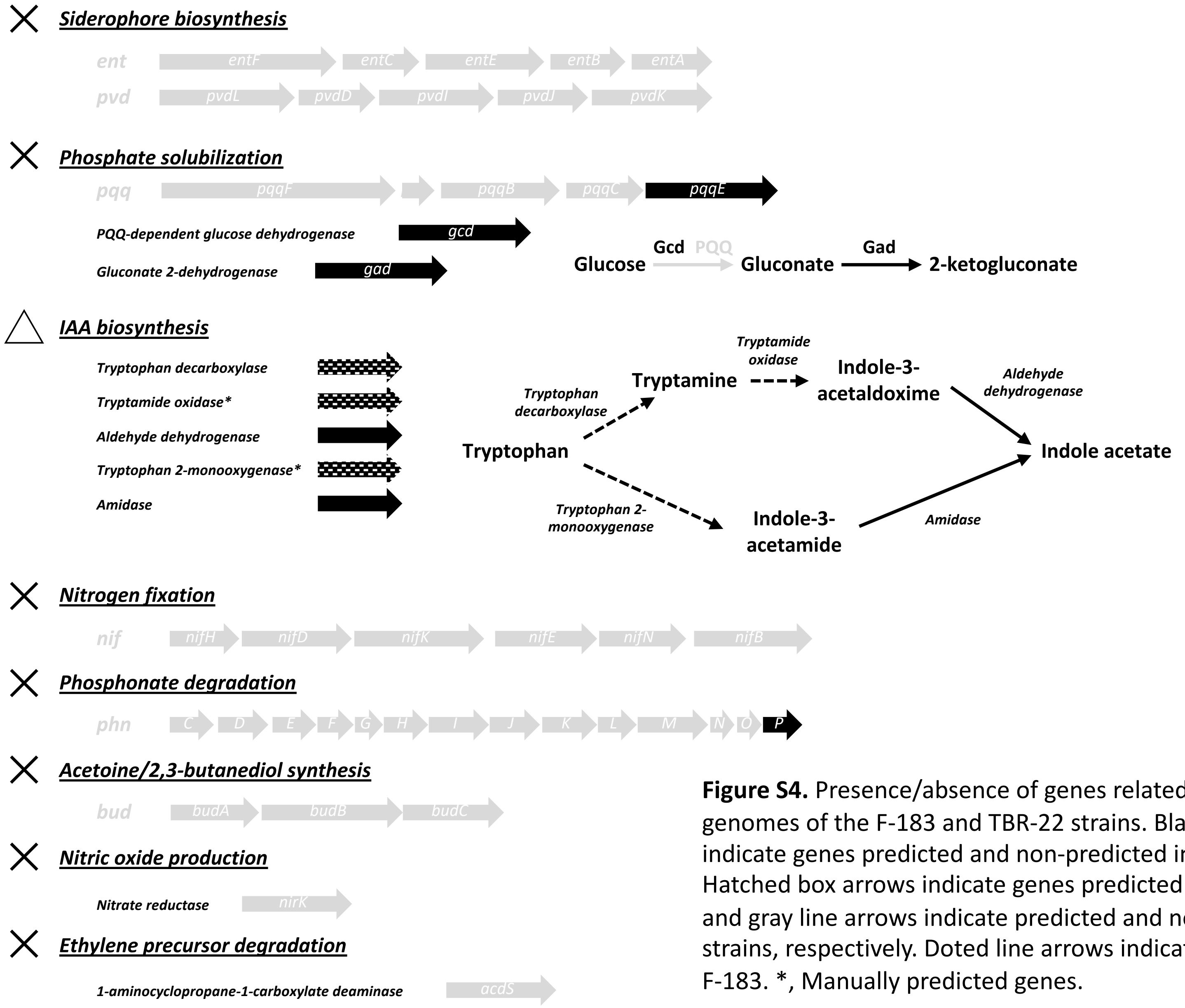

Figure S4. Presence/absence of genes related to typical PGP traits in the genomes of the F-183 and TBR-22 strains. Black and gray box arrows indicate genes predicted and non-predicted in two genomes, respectively. Hatched box arrows indicate genes predicted in the F-183 genome. Black and gray line arrows indicate predicted and non-predicted reactions in both strains, respectively. Doted line arrows indicate reactions predicted in strain F-183. *, Manually predicted genes.

Supplementary Figure S5

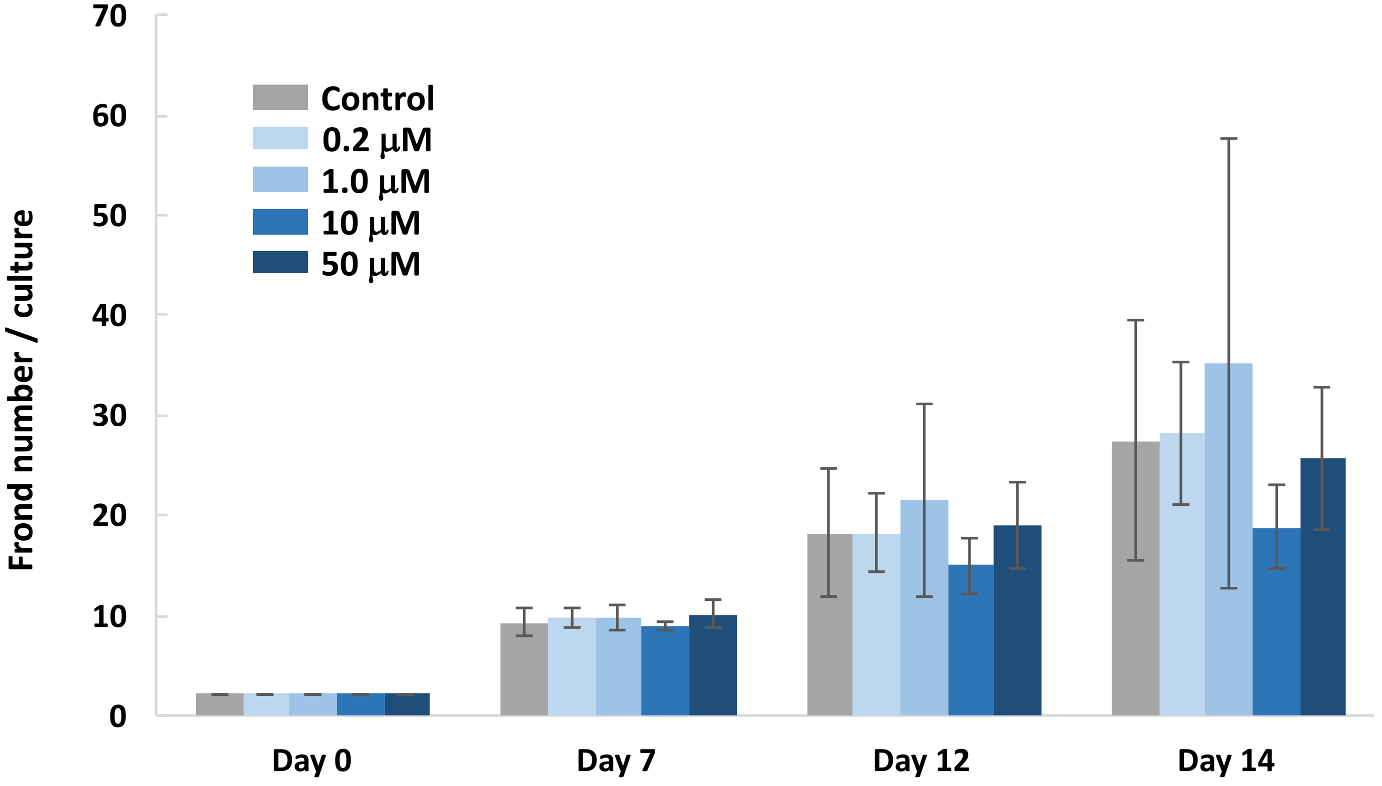

Figure S5. Effect of IAA on duckweed growth. *L. aequinoctialis* was cultivated in 50 ml of mHoagland medium supplemented with IAA at indicated concentrations (N=6). No significant differences detected among treatments in each day.

Supplementary Figure S6

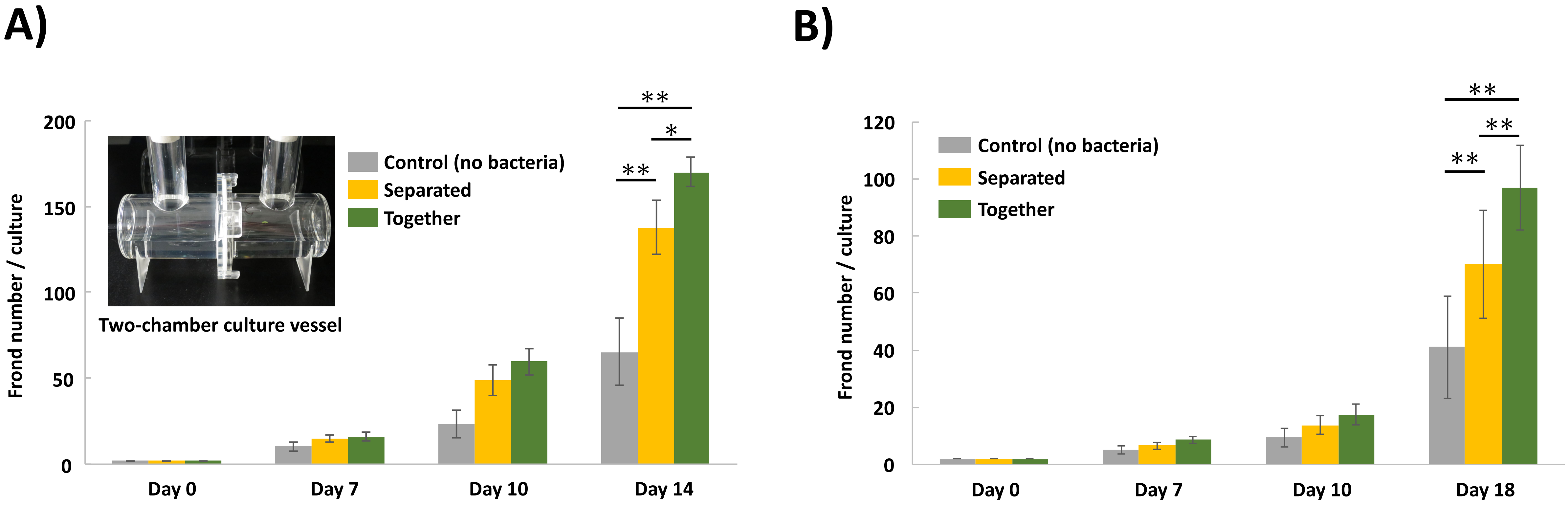

Figure S6. PGP effect of the F-183 and TBR-22 strains on duckweed in membrane-separated co-cultivation. *L. aequinoctialis* was cocultivated with A) strain F-183 or B) strain TBR-22 in two-chamber dialysis culture vessels (Nihon Pall Ltd. Japan). Two chambers were separated by 0.2 um-pore size membrane, and each chamber contained 30 ml of mHoagland medium. *L. aequinoctialis* and strains F-183 or TBR-22 were cultured in the same chamber (Together) or in each membrane-separate chamber (Separated). In control cultures, one chamber was inoculated with *L. aequinoctialis* and another chamber was medium only. F-183 cells were harvested from 10-day cultures (OD_{600}=0.27) by centrifugation, washed once with mHoagland medium, and dispersed in the medium in the culture chamber (final OD_{600}=0.27). N=4; *, P<0.05; **, P<0.01. TBR-22 cells were harvested from 16- or 20-day cultures (OD_{600}=0.12 or 0.06) by centrifugation, washed once with mHoagland medium, and dispersed in the medium in the culture chamber (final OD_{600}=0.17 and 0.2). N=8; **, P<0.01.

Supplementary Table S1

Table S1. Plant growth promotion effects of *Acidobacteria* strains F-183 and TBR-22 on duckweed members of subfamily *Lemnoideae.*
(mean frond numbers at day 14 [n=3, with SD]).

	Spirodela polyrhiza	*Landoltia punctata*	*Lemna minor*	*Wolffia arrhiza*	*Wolffia globosa*
F-183	60.0 (±3.6)	57.0 (±5.7)	61.0 (±2.9)	55.3 (±1.9)	94.0 (±4.1)
control	30.0 (±4.3)	22.3 (±0.5)	31.7 (±1.3)	30.7 (±8.7)	18.3 (±4.0)
**F-183/control**	**2.0****	**2.6****	**1.9****	**1.8***	**5.1****
TBR-22	73.7 (±12)	70.0 (±1.7)	60.0 (±2.2)	52.7 (±0.5)	69.3 (±2.6)
control	33.3 (±2.5)	26.3 (±2.1)	25.0 (±5.0)	19.0 (±4.5)	18.0 (±0.8)
**TBR-22/control**	**2.2****	**2.6****	**2.4****	**2.8****	**3.9****

**, $p<0.01$; *, $p<0.05$

Supplementary Table S2

Table S2. Predicted genes related to PGP traits in the genome of the F-183 strain

Locus tag	Gene*	EC_number*	COG*	Product*	KO**	Definition**
Iron acquision						
Siderophore biosynthesis						
None						
Phosphate solubilization						
PQQ biosynthesis						
F183_03770	*pqqE_1*	1.21.98.4		PqqA peptide cyclase	K06139	pqqE; PqqA peptide cyclase [EC:1.21.98.4]
F183_13510	*pqqE_2*	1.21.98.4		PqqA peptide cyclase		
Glucose dehydrogenase						
F183_37620	*gcd*	1.1.5.2	COG4993	Quinoprotein glucose dehydrogenase	K00117	gcd; quinoprotein glucose dehydrogenase [EC:1.1.5.2]
F183_30740	*quiA_1*	1.1.5.8	COG4993	Quinate/shikimate dehydrogenase (quinone)	K00117	gcd; quinoprotein glucose dehydrogenase [EC:1.1.5.2]
F183_33560	*quiA_2*	1.1.5.8	COG4993	Quinate/shikimate dehydrogenase (quinone)	K00117	gcd; quinoprotein glucose dehydrogenase [EC:1.1.5.2]
F183_39650	*bamB_14*			Outer membrane protein assembly factor BamB	K00117	gcd; quinoprotein glucose dehydrogenase [EC:1.1.5.2]
Gluconate 2-dehydrogenase						
F183_07970				hypothetical protein	K06152	E1.1.99.3G; gluconate 2-dehydrogenase gamma chain [EC:1.1.99.3]
F183_53030				hypothetical protein	K06152	E1.1.99.3G; gluconate 2-dehydrogenase gamma chain [EC:1.1.99.3]
IAA biosynthesis						
F183_53730	*adh_2*	1.2.1.3	COG1012	Aldehyde dehydrogenase	K00138	aldB; aldehyde dehydrogenase [EC:1.2.1.-]
F183_29190	*aam*	3.5.1.13	COG0154	Acylamidase	K01426	E3.5.1.4; amidase [EC:3.5.1.4]
F183_01440	*amiD*	3.5.1.4	COG0154	Putative amidase AmiD		
F183_52410		4.1.1.105	COG0076	Tryptophan decarboxylase	K01593	DDC; aromatic-L-amino-acid/L-tryptophan decarboxylase [EC:4.1.1.28 4.1.1.105]
F183_18990		4.1.1.105	COG0076	Tryptophan decarboxylase		
Nitrogen fixation						
Nitrogenase complex						
None						

* Annotation by Prokka, ** Annotation by BlastKOALA

Supplementary Table S3

Table S3. Predicted genes related to PGP traits in the genome of the TBR-22 strain

Locus tag	Gene*	EC_number*	COG*	Product*	KO**	Definition**
Iron acquision						
Siderophore biosynthesis						
None						
Phosphate solubilization						
PQQ biosynthesis						
TBR22_22110	*pqqE_1*			Coenzyme PQQ synthesis protein E	K06937	
TBR22_24790	*pqqE_2*			Coenzyme PQQ synthesis protein E		
TBR22_34680	*pqqE_3*			Coenzyme PQQ synthesis protein E		
Glucose dehydrogenase						
TBR22_12440	*gcd_1*	1.1.5.2	COG4993	Quinoprotein glucose dehydrogenase	K00117	gcd; quinoprotein glucose dehydrogenase [EC:1.1.5.2]
TBR22_15650	*gcd_2*	1.1.5.2	COG4993	Quinoprotein glucose dehydrogenase	K00117	gcd; quinoprotein glucose dehydrogenase [EC:1.1.5.2]
TBR22_15660	*gcd_3*	1.1.5.2	COG4993	Quinoprotein glucose dehydrogenase	K00117	gcd; quinoprotein glucose dehydrogenase [EC:1.1.5.2]
TBR22_32530	*gcd_4*	1.1.5.2	COG4993	Quinoprotein glucose dehydrogenase	K00117	gcd; quinoprotein glucose dehydrogenase [EC:1.1.5.2]
TBR22_41070	*quiA_1*	1.1.5.8	COG4993	Quinate/shikimate dehydrogenase (quinone)	K00117	gcd; quinoprotein glucose dehydrogenase [EC:1.1.5.2]
TBR22_42110	*quiA_2*	1.1.5.8	COG4993	Quinate/shikimate dehydrogenase (quinone)	K00117	gcd; quinoprotein glucose dehydrogenase [EC:1.1.5.2]
TBR22_43950	*gcdC*	4.1.1.70	COG0511	Glutaconyl-CoA decarboxylase subunit gamma		
TBR22_40480				hypothetical protein	K00117	gcd; quinoprotein glucose dehydrogenase [EC:1.1.5.2]
Gluconate 2-dehydrogenase						
TBR22_21570		1.1.99.3		Gluconate 2-dehydrogenase subunit 3	K06152	E1.1.99.3G; gluconate 2-dehydrogenase gamma chain [EC:1.1.99.3]
TBR22_13060		1.1.99.3		Gluconate 2-dehydrogenase cytochrome c subunit	K23275	fdhC; fructose 5-dehydrogenase cytochrome subunit
IAA biosynthesis						
TBR22_00130	*acoD*	1.2.1.3	COG1012	Acetaldehyde dehydrogenase 2	K00138	aldB; aldehyde dehydrogenase [EC:1.2.1.-]
TBR22_02620	*amiD*	3.5.1.4	COG0154	Putative amidase AmiD		
TBR22_27620				hypothetical protein	K01426	E3.5.1.4; amidase [EC:3.5.1.4]
Nitrogen fixation						
Nitrogenase complex						
None						

* Annotation by Prokka, ** Annotation by BlastKOALA

Supplementary Table S4

Table S4. Predicted genes related to PGP traits in the genomes of the F-183 and TBR-22 strains

Locus tag	Gene*	EC_number*	COG*	Product*	KO**	Definition**
phosphonate metabolism	**(*phn*)**					
F183_38540	*phnP*	3.1.4.55	COG1235	Phosphoribosyl 1,2-cyclic phosphate phosphodiesterase	K06167	phnP; phosphoribosyl 1,2-cyclic phosphate phosphodiesterase [EC:3.1.4.55]
F183_54190	*yycJ*	3.-.-.-	COG1235	Putative metallo-hydrolase YycJ		
TBR22_32620	*phnP*	3.1.4.55	COG1235	Phosphoribosyl 1,2-cyclic phosphate phosphodiesterase	K06167	phnP; phosphoribosyl 1,2-cyclic phosphate phosphodiesterase [EC:3.1.4.55]
Acetoine/2,3-butanediol synthesis	**(*budB*)**					
F183_27870	*ilvB*	2.2.1.6	COG0028	Acetolactate synthase large subunit	K01652	E2.2.1.6L; acetolactate synthase I/II/III large subunit [EC:2.2.1.6]
F183_27880	*ilvH*	2.2.1.6	COG0440	Acetolactate synthase small subunit	K01653	E2.2.1.6S; acetolactate synthase I/III small subunit [EC:2.2.1.6]
F183_33220	*ilvG*	2.2.1.6		Acetolactate synthase isozyme 2 large subunit	K01652	E2.2.1.6L; acetolactate synthase I/II/III large subunit [EC:2.2.1.6]
F183_15880	*xsc*	2.3.3.15		Sulfoacetaldehyde acetyltransferase	K01652	E2.2.1.6L; acetolactate synthase I/II/III large subunit [EC:2.2.1.6]
TBR22_22350	*ilvH*	2.2.1.6	COG0440	Putative acetolactate synthase small subunit	K01653	E2.2.1.6S; acetolactate synthase I/III small subunit [EC:2.2.1.6]
TBR22_22360	*ilvB*	2.2.1.6	COG0028	Acetolactate synthase large subunit	K01652	E2.2.1.6L; acetolactate synthase I/II/III large subunit [EC:2.2.1.6]

* Annotation by Prokka, ** Annotation by BlastKOALA
